# Global Patterns in Human Mitochondrial DNA and Y-Chromosome Variation Caused by Spatial Instability of the Local Cultural Processes

**DOI:** 10.1371/journal.pgen.0020053

**Published:** 2006-04-14

**Authors:** Vikrant Kumar, Banrida T Langstieh, Komal V Madhavi, Vegi M Naidu, Hardeep Pal Singh, Silpak Biswas, Kumarasamy Thangaraj, Lalji Singh, B. Mohan Reddy

**Affiliations:** 1 Biological Anthropology Unit, Indian Statistical Institute, Hubsiguda, Hyderabad, India; 2 Department of Anthropology, North Eastern Hill University, Shillong, India; 3 GSF Hematologikum, Medizinische Klink III, Klinikum Grosshadern, München, Germany; 4 Kallam Anji Reddy Molecular Genetics Laboratory, L.V. Prasad Eye Institute, L.V. Prasad Marg, Banjara Hills, India; 5 Centre for Cellular and Molecular Biology, Hyderabad, India; Wellcome Trust Sanger Institute, United Kingdom

## Abstract

Because of the widespread phenomenon of patrilocality, it is hypothesized that Y-chromosome variants tend to be more localized geographically than those of mitochondrial DNA (mtDNA). Empirical evidence confirmatory to this hypothesis was subsequently provided among certain patrilocal and matrilocal groups of Thailand, which conforms to the isolation by distance mode of gene diffusion. However, we expect intuitively that the patterns of genetic variability may not be consistent with the above hypothesis among populations with different social norms governing the institution of marriage, particularly among those that adhere to strict endogamy rules. We test the universality of this hypothesis by analyzing Y-chromosome and mtDNA data in three different sets of Indian populations that follow endogamy rules to varying degrees. Our analysis of the Indian patrilocal and the matrilocal groups is not confirmatory to the sex-specific variation observed among the tribes of Thailand. Our results indicate spatial instability of the impact of different cultural processes on the genetic variability, resulting in the lack of universality of the hypothesized pattern of greater Y-chromosome variation when compared to that of mtDNA among the patrilocal populations.

## Synopsis

In most human societies, women traditionally move to their husband's home after marriage, and these societies are thus “patrilocal,” but in a few “matrilocal” societies, men move to their wife's home. These social customs are expected to influence the patterns of genetic variation. They should lead to a localization of male-specific Y-chromosomal variants and wide dispersal of female-specific mitochondrial DNA variants in patrilocal societies and vice versa in matrilocal societies. These predicted patterns have indeed been observed in previous studies of populations from Thailand. Indian societies, however, are endogamous, so marriage should always take place within a population, and these different patterns of genetic variation should not build up. The authors have now analyzed ten patrilocal and five matrilocal Indian populations, and find that there is indeed little difference between the patrilocal and matrilocal societies. The authors therefore conclude that patterns of genetic variation in humans are not universal, but depend on local cultural practices.

## Introduction

The genetic patterns in human societies are often fashioned by their cultural practices. For example, it has been hypothesized that due to widespread phenomenon of patrilocality (a pattern of residence where the female spouse after marriage resides in the in-law's house) Y-chromosome variants tend to be more localized geographically than those of mitochondrial DNA (mtDNA) and the autosomes, and therefore high degree of inter-population genetic differences have been observed for the Y chromosome compared to the mtDNA [1–4]. Due to movement of females in patrilocal groups, the mtDNA diversity is assumed to be high within the populations and low between the populations, whereas the Y-chromosome diversity will be relatively low within the groups and high between the groups. This pattern is expected to be reversed in case of the matrilocal groups (a pattern of residence where the males after marriage reside in the in-law's house). Empirical evidence confirmatory to this hypothesis was subsequently provided by Oota et al. [5] among the three patrilocal and three matrilocal groups of Thailand. They found genetic diversity to be strikingly correlated with residence patterns suggesting the role of sex-specific patterns of migration in influencing the genetic patterns. In contrast, few other studies at the regional scale [6–8] show similar levels of differentiation for maternal and paternal lineages. Therefore, the patterns of genetic diversity at the local level may not reflect at the global scale, which is essentially an artifact of the sum total of differing local patterns. Concurrently, in a global survey, Wilder et al. [9] could not detect the signature of a higher inter-population migration rate for females than for males. This is interpreted as due to lack of geographic stability of the behavioral customs of individual populations necessary to influence global genetic patterning. The norms governing the institution of marriage vary enormously among human populations of different regions or cultures [10–12], and different forms of social organization can impact patterns and levels of genetic diversity [13,14]. Therefore, the universality of the above hypothesis, i.e., the pattern of genetic variation vis-à-vis the residence pattern of spouses, is in question.

**Figure 1:**
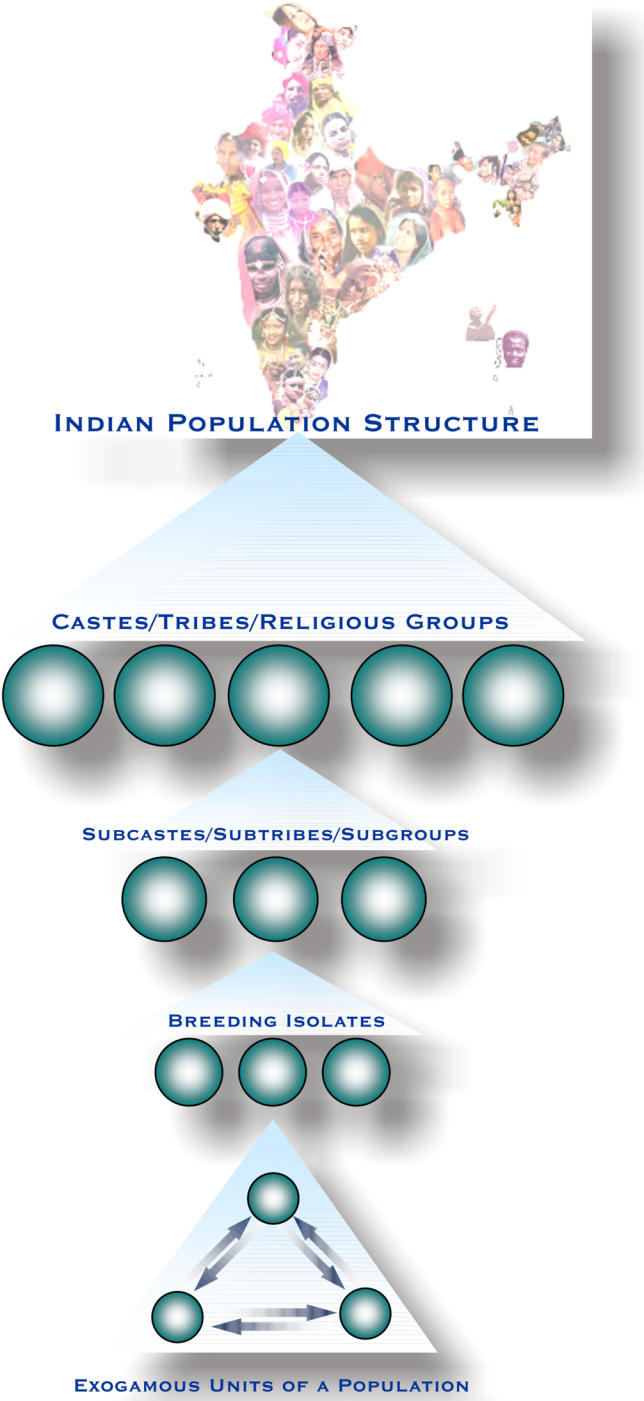
Schematic Representation of Indian Population Structure Characterized by Movement of Spouses Only within but Not among the Endogamous Groups Each circle represents a population and its size represents the hierarchy. While the populations until the breeding isolates are all endogamous, the exogamous units refer to clans/lineages within a breeding isolate/population.

Implicit in the above hypothesis is the assumption that the population boundaries are permeable, permitting male/female spouses to move across their respective populations and become part of the gene pool of the new population to which the other spouse belongs. Only in such a scenario can the expectations of the above hypothesis hold, either in patrilocal or matrilocal societies. This situation, broadly speaking, approximates to isolation by distance mode of gene diffusion. On the other hand, for populations bound by rigid endogamy rules with their boundaries absolutely impermeable, neither patrilocality nor matrilocality can make any difference to their genetic variability, be it Y-chromosome or mtDNA, since the movement is restricted to within a population. The Indian subcontinent with its unique population structure and strictly defined endogamous castes, tribes, and religious groups is a case in point (Figure 1). The marriage interactions are restricted within an endogamous population consisting of the number of exogamous units/clans between which marriages take place. We directly test the universality of the hypothesis delineated above and attempt to assess the spatial stability of the local cultural processes necessary to influence global patterning in two stages. In the first stage, we analyzed Y-chromosome short tandem repeat (Y-STR) and mtDNA hyper variable segment 1 (HVS1) sequence data from two groups of Indian tribes, comprised of five populations each, belonging to a broad linguistic family and with similar socio-economic status. The genetic data were obtained from the same set of populations and individuals making it appropriate for comparison. The populations included in this study are Maram, Khynriam, Pnar, Bhoi, and WarKhasi, the five matrilocal Khasi tribes of Meghalaya in the Northeastern part of India; and Asur, Bhumij, Kharia, Munda, and Santhal, the five patrilocal Mundari tribes of Eastern India, who along with the matrilocal Khasis, belong to the broad Austro-Asiatic linguistic family. At the second stage, to gauge the consistency in the genetic patterns within broad regional or cultural context, the same set of genetic data were generated on the five Dravidian language-speaking patrilocal caste populations from Andhra Pradesh (Akutota, Kapu, Panta, Pokanati, and Vanne) of Southern India and compared with the Austro-Asiatic matrilocal tribes.

The structure of populations considered in this study is characterized by numerous endogamous groups cohabiting as islands with no or negligible gene flow between them. Therefore, as the marital boundary of each population is impermeable, we intuitively expect that the pattern of genetic variability may not strictly follow the expectations of the aforesaid hypothesis, either in patrilocal or matrilocal groups. All three groups of populations have contiguous geographic distribution in their respective areas, which provide opportunity for exchange of mates, if the social norms permit, thus providing ideal study frame.

## Results

**Figure 2:**
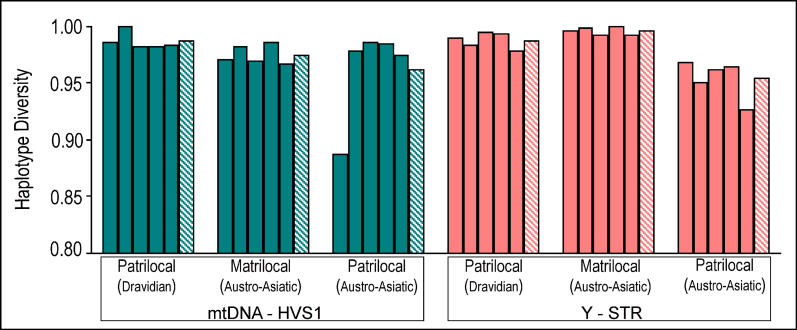
Haplotype Diversity in mtDNA (Green) and Y-STR (Pink) and Their Mean (Shaded Bar) in Five Dravidian and Five Austro-Asiatic Patrilocal and Five Austro-Asiatic Matrilocal Populations From left to right, the Dravidian patrilocal groups (mtDNA sample size and Y-STR sample size) are Akhutota (32, 21), Kapu (22,16), Panta (37, 21), Pokanati (59, 25), and Vanne (32, 23); the Austro-Asiatic matrilocal groups are Maram (72, 58), Khynriam (95, 82), Pnar (69, 40), Bhoi (34, 30), and WarKhasi (31, 23); the Austro-Asiatic patrilocal groups are Asur (30, 28), Bhumij (40, 39), Kharia (21, 13), Munda (23, 23), and Santhal (39, 38).

Within-group mtDNA diversity (Figure 2) is similar (Mann-Whitney U test, *p* = 0.690) for matrilocal Khasi tribes (0.975) versus patrilocal Mundari tribes (0.962), although the mean within-group Y-chromosome diversity of patrilocal Mundari tribal groups (0.954) is significantly lower (Mann-Whitney U test, *p* = 0.008) when compared with matrilocal Khasi (0.995). However, when we compare the patrilocal Dravidian caste groups with the matrilocal Khasi tribes we found similar and non-significant difference in the level of within-group diversity for both mtDNA (*p* = 0.056) and Y-chromosome (*p* = .095). The average values of genetic distance (Table 1) reflecting inter-group diversity (although smaller for mtDNA and larger for Y-chromosome among patrilocal Mundari groups than for matrilocal Khasi groups) are not statistically significantly different. Likewise, the average genetic distances in the Dravidian patrilocal groups are smaller for mtDNA and larger for Y-chromosome but not significantly so when compared with the matrilocal Khasi groups.

**Table 1:**
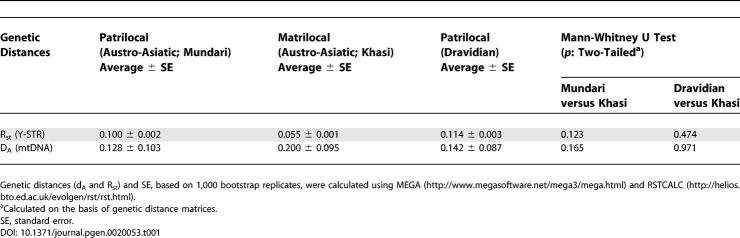
Average Genetic Distance and Their Standard Error Based on mtDNA HVS1 and Y-STR among the Matrilocal and Patrilocal Groups

The index of probability of identity, which gives a quantitative measure of haplotype sharing between a pair of populations, further suggests, as against the hypothesis, that the degree of Y-chromosome haplotype sharing (Table 2), although not significant, is substantially higher among the patrilocal Mundari groups when compared with the Matrilocal Khasi tribes, whereas the degree of mtDNA haplotype sharing is almost identical for both groups. On the other hand, we observe a very low level of mtDNA haplotype sharing among the patrilocal Dravidian groups compared with the matrilocal Khasi groups, while the level of Y-chromosome haplotype sharing is similar for both the groups. As per the hypothesis, a relatively lower degree of mtDNA haplotype sharing and greater degree of Y-chromosome haplotype sharing is expected among the matrilocal groups compared with the patrilocal groups. Overall, the results are not consistent with the universality of the hypothesis in question.

**Table 2:**
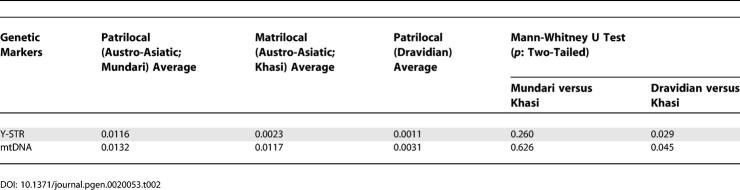
Index of Probability of Identity Based on mtDNA HVS1 and Y-STR among the Patrilocal and Matrilocal Groups

## Discussion

The foregoing analysis of the results does not reflect higher migration rate of females and males, respectively, in the patrilocal and matrilocal populations, suggesting that the pattern of residence of the spouses has no bearing on the mtDNA and Y-chromosome variability in the populations, in which sex-specific migrations implicit in the hypothesis are confined within the endogamous groups and do not usually transect the caste/tribal boundaries. However, a weak and non-significant trend of greater inter-group variation in Y-chromosome and lower variation in mtDNA in case of patrilocal groups, and greater mtDNA and lower Y-chromosome inter-group variation in matrilocal groups, which is consistent with the hypothesis, is observed. Nevertheless, the magnitude of differences, either intra- or inter-population observed in our study, are substantially smaller than what has been observed by Oota et al. [5] in Thailand, despite a relatively small number of samples and populations. The non-significant differences in the mean values of the genetic distances could have been due to two reasons: (1) either to small sample size; hence lacking sufficient power to correctly reject the null hypothesis, or (2) to small number of Y-STRs, which may not have adequate resolution. Therefore, we calculated power of the Mann-Whitney U test for the given sample sizes in the study and the results suggest that the test has > 99% power, even at alpha = 0.001, both for mtDNA and Y-chromosome. Additional analysis based on 15 Y-STRs suggests, contrary to the hypothesis, that the average genetic distance among the patrilocal groups was quite low (0.0469 ± 0.0009), albeit non-significantly (*p* = 0.1), as compared with the matrilocal groups (0.1024 ± 0.0024). Therefore, the hypothesized correlation of genetic diversity with the sex-specific migration patterns may not be applicable to the Indian situation, although it is observed elsewhere in certain populations whose marital boundaries are probably permeable.

One of the questions raised by Wilder et al. [9] is the extent to which local cultural practices influence genetic patterns at the regional and global scale. The groups we have considered in the present study have different cultural norms governing the rules of marriages compared to those studied by Oota et al. [5]; hence we find variation in the genetic patterns. Even within India, we find variation in the pattern depending on whether we compare the matrilocal Khasi tribes with the patrilocal Mundari tribal groups or with the patrilocal Dravidian caste groups. For example, the index of probability of identity shows very low values for both mtDNA and Y-chromosome haplotype sharing among the Dravidian castes when compared with the Austro-Asiatic tribes, either Mundari or Khasi (Table 2). This pattern is observed because the caste populations of India are considered to follow endogamy very strictly; hence their marital boundaries are highly rigid compared with the marital boundaries of the Indian tribes, particularly from Northeast India, suggesting the impact of varying cultural practices pertaining particularly to marriage, resulting in variable genetic patterns. Results of our study taken together with the previous studies, that have [6–8] or have not [1,3–5] detected sex-specific migration, suggest that the local cultural processes do not have spatial stability required to influence global patterning. Perhaps due to this, Wilder et al. [9] did not observe higher migration rates of females vis-à-vis males at the continental level, although most of the populations of the world follow patrilocality [15]. Therefore, the hypothesis of greater Y-chromosome vis-à-vis mtDNA variability due to patrilocality is not universal, as it can only be selectively applicable to populations with cultural norms that permit inter-group marriages; not to, for example, highly endogamous Indian populations. Pertinent to this are the two recent large-scale Indian studies [16,17] wherein the lack of spatial structure in the quantitative biological variables—anthropometry and dermatoglyphics— and traditional genetic markers was inferred to be consistent with population structure characterized by numerous endogamous groups cohabiting as islands with no or negligible gene flow between them; the monotonic decline in the spatial autocorrelation expected under the model of contiguous diffusion of genes is not evident in those data.

## Materials and Methods

Blood samples from 636 individuals belonging to 15 populations were obtained for the above populations during 2000–2003 with informed written consent; DNA was extracted. The names of the populations along with their sample size are given in Figure 2. We analyzed 350 base pairs of the HVS1 of the mtDNA control region corresponding to positions 16050–16400 and six Y-STR loci (DYS19, DYS389I, DYS389b, DYS390, DYS391, and DYS393). Allele length for DYS389b was obtained by subtracting the allele length of DYS389I from DYS389II. The HVS1 sequences have been submitted to GenBank and are also available from the authors, as are the Y-STR data. To measure within-group variability we estimated haplotype diversity [18] for the HVS1 sequences and Y-STR haplotypes (Table S1), and calculated d_A_ distances [19] for the HVS1 sequences using the number of different sites model, and R_ST_ for the Y-STR haplotypes [20] as measures of between-group diversity. Further, we computed an index of probability of identity [21], which gives a quantitative measure of haplotype sharing between a pair of populations. To ascertain, for the given sample sizes, that the test has enough power at alpha = 0.05– 0.001, we computed power required for the Mann-Whitney U test. For this purpose, we decreased the sample sizes by 15% and used this sample size to compute power required for a *t*-test. This rule is based on the lower bound for the asymptotic relative efficiency (ARE) of the Mann-Whitney U test versus the *t*-distribution, which is 0.864. This says that no matter what the distribution is, the ARE of the Mann-Whitney U test can never be worse than 0.864 for a reasonable broad class of probability distributions. Inverting that gives an increase in the sample size by a factor of 1.157, and therefore the sample sizes were reduced by 15% [22]. To increase the resolution, in addition to the six Y-STRs, we typed nine more Y-STRs (DYS388, DYS426, DYS437, DYS438, DYS439, DYS447, DYS448, DYS460, and H4; Table S2) in three populations each of Mundari patrilocal groups (Bhumij, Munda, and Santhal) and Khasi matrilocal groups (Khynriam, Maram, and Pnar) and recomputed genetic distances based on 15 Y-STR loci.

## Supporting Information

Table S1 Y-Chromosome Haplotypes Based on Six Y-STRs for 15 Populations (946 KB DOC)

Table S2 Y-Chromosome Haplotypes Based on Nine Y-STRs for Six Populations (387 KB DOC)

### Accession Numbers

The GenBank (http://www.ncbi.nlm.nih.gov) accession numbers for the sequence discussed in this paper are HVS1 (AY72095–AY721592).

## Abbreviations

HVS1: hyper variable segment 1
mtDNA: mitochondrial DNA
Y-STR: Y-chromosome short tandem repeat
