# Correction: Global Patterns in Human Mitochondrial DNA and Y-Chromosome Variation Caused by Spatial Instability of the Local Cultural Processes

**DOI:** 10.1371/journal.pgen.0020095

**Published:** 2006-06-30

**Authors:** Vikrant Kumar, Banrida T Langstieh, Komal V Madhavi, Vegi M Naidu, Hardeep Pal Singh, Silpak Biswas, Kumarasamy Thangaraj, Lalji Singh, B. Mohan Reddy

In *PLoS Genetics,* vol 2, issue 4, DOI:  10.1371/journal.pgen.0020053

In the Supporting Information section, the sentence “The GenBank (http://www.ncbi.nlm.nih.gov) accession numbers for the sequence discussed in this paper are HVS1 (AY72095-AY721592)” should be “The GenBank (http://www.ncbi.nlm.nih.gov) accession numbers for the sequence discussed in this paper are HVS1 (AY720957-AY721592).”

