# Supplementary material for: Global Patterns in Human Mitochondrial DNA and Y-Chromosome Variation Caused by Spatial Instability of the Local Cultural Processes
Source: PLoS Genet. 2006 Apr 14;2(4):e53. doi: 10.1371/journal.pgen.0020053 (PMC1435684; doi:10.1371/journal.pgen.0020053)
Supplement: Supplementary file 1 [file pgen.0020053.st001.doc]

Supplementary Table 1: Y-chromosome Haplotypes based on 6 Y-STRs for 15 Populations

|  | **19** | **389I** | **389b** | **390** | **391** | **393** |
| --- | --- | --- | --- | --- | --- | --- |
| **Mundari** |  |  |  |  |  |  |
| ***Asur*** |  |  |  |  |  |  |
| Sample 1 | 15 | 14 | 17 | 25 | 9 | 14 |
| Sample 2 | 15 | 14 | 16 | 25 | 10 | 14 |
| Sample 3 | 15 | 13 | 16 | 24 | 9 | 12 |
| Sample 4 | 15 | 13 | 15 | 25 | 9 | 14 |
| Sample 5 | 15 | 14 | 17 | 25 | 9 | 13 |
| Sample 6 | 15 | 14 | 18 | 25 | 9 | 12 |
| Sample 7 | 15 | 14 | 17 | 25 | 9 | 12 |
| Sample 8 | 15 | 13 | 16 | 22 | 10 | 11 |
| Sample 9 | 15 | 13 | 16 | 24 | 9 | 12 |
| Sample 10 | 15 | 13 | 16 | 24 | 9 | 12 |
| Sample 11 | 15 | 13 | 16 | 22 | 10 | 12 |
| Sample 12 | 15 | 13 | 16 | 24 | 9 | 12 |
| Sample 13 | 15 | 14 | 17 | 25 | 9 | 12 |
| Sample 14 | 15 | 13 | 16 | 24 | 9 | 12 |
| Sample 15 | 15 | 13 | 15 | 25 | 9 | 12 |
| Sample 16 | 15 | 13 | 15 | 25 | 9 | 12 |
| Sample 17 | 15 | 12 | 16 | 26 | 9 | 12 |
| Sample 18 | 15 | 13 | 16 | 24 | 9 | 12 |
| Sample 19 | 15 | 13 | 16 | 24 | 9 | 12 |
| Sample 20 | 16 | 12 | 16 | 25 | 9 | 12 |
| Sample 21 | 16 | 12 | 16 | 25 | 9 | 12 |
| Sample 22 | 15 | 13 | 16 | 25 | 10 | 12 |
| Sample 23 | 16 | 12 | 16 | 25 | 9 | 12 |
| Sample 24 | 16 | 12 | 16 | 25 | 9 | 11 |
| Sample 25 | 16 | 12 | 16 | 25 | 9 | 11 |
| Sample 26 | 15 | 12 | 16 | 25 | 9 | 12 |
| Sample 27 | 15 | 13 | 16 | 25 | 11 | 12 |
| Sample 28 | 15 | 13 | 18 | 25 | 10 | 13 |
|  |  |  |  |  |  |  |
| ***Bhumij*** |  |  |  |  |  |  |
| Sample 1 | 15 | 13 | 15 | 26 | 9 | 12 |
| Sample 2 | 15 | 15 | 16 | 25 | 9 | 12 |
| Sample 3 | 15 | 13 | 15 | 23 | 9 | 12 |
| Sample 4 | 15 | 13 | 15 | 24 | 9 | 12 |
| Sample 5 | 15 | 13 | 16 | 26 | 9 | 12 |
| Sample 6 | 15 | 14 | 16 | 25 | 9 | 12 |
| Sample 7 | 15 | 15 | 16 | 25 | 9 | 12 |
| Sample 8 | 15 | 13 | 16 | 24 | 8 | 12 |
| Sample 9 | 16 | 12 | 15 | 23 | 9 | 12 |
| Sample 10 | 15 | 13 | 15 | 24 | 9 | 12 |
| Sample 11 | 15 | 13 | 15 | 24 | 9 | 12 |
| Sample 12 | 15 | 14 | 17 | 22 | 9 | 12 |
| Sample 13 | 15 | 13 | 15 | 24 | 9 | 12 |
| Sample 14 | 17 | 14 | 17 | 25 | 10 | 12 |
| Sample 15 | 16 | 12 | 15 | 23 | 9 | 12 |
| Sample 16 | 15 | 14 | 17 | 22 | 9 | 12 |
| Sample 17 | 14 | 13 | 15 | 22 | 9 | 12 |
| Sample 18 | 16 | 12 | 15 | 23 | 9 | 12 |
| Sample 19 | 16 | 13 | 16 | 26 | 9 | 12 |
| Sample 20 | 15 | 14 | 17 | 22 | 9 | 12 |
| Sample 21 | 16 | 13 | 16 | 26 | 9 | 12 |
| Sample 22 | 16 | 13 | 16 | 26 | 9 | 12 |
| Sample 23 | 15 | 13 | 15 | 24 | 9 | 12 |
| Sample 24 | 16 | 13 | 16 | 25 | 9 | 12 |
| Sample 25 | 15 | 14 | 17 | 22 | 9 | 12 |
| Sample 26 | 16 | 14 | 17 | 26 | 10 | 12 |
| Sample 27 | 15 | 14 | 17 | 22 | 9 | 12 |
| Sample 28 | 15 | 14 | 17 | 22 | 9 | 12 |
| Sample 29 | 15 | 14 | 16 | 23 | 9 | 12 |
| Sample 30 | 15 | 12 | 16 | 26 | 9 | 12 |
| Sample 31 | 16 | 13 | 16 | 24 | 9 | 12 |
| Sample 32 | 15 | 13 | 15 | 26 | 9 | 12 |
| Sample 33 | 15 | 14 | 16 | 26 | 9 | 12 |
| Sample 34 | 16 | 13 | 17 | 24 | 9 | 12 |
| Sample 35 | 15 | 14 | 17 | 22 | 9 | 12 |
| Sample 36 | 14 | 12 | 17 | 23 | 10 | 12 |
| Sample 37 | 15 | 13 | 16 | 26 | 9 | 12 |
| Sample 38 | 15 | 14 | 16 | 25 | 9 | 12 |
| Sample 39 | 14 | 12 | 17 | 24 | 10 | 12 |
|  |  |  |  |  |  |  |
| ***Kharia*** |  |  |  |  |  |  |
| Sample 1 | 15 | 13 | 15 | 25 | 9 | 12 |
| Sample 2 | 16 | 14 | 16 | 22 | 9 | 12 |
| Sample 3 | 16 | 14 | 16 | 22 | 9 | 12 |
| Sample 4 | 15 | 14 | 17 | 25 | 10 | 12 |
| Sample 5 | 15 | 14 | 16 | 23 | 9 | 12 |
| Sample 6 | 15 | 13 | 17 | 26 | 10 | 12 |
| Sample 7 | 15 | 13 | 16 | 25 | 10 | 12 |
| Sample 8 | 15 | 12 | 16 | 25 | 9 | 12 |
| Sample 9 | 14 | 14 | 16 | 22 | 9 | 12 |
| Sample 10 | 16 | 13 | 16 | 24 | 9 | 12 |
| Sample 11 | 17 | 13 | 18 | 26 | 10 | 12 |
| Sample 12 | 15 | 13 | 17 | 24 | 9 | 12 |
| Sample 13 | 15 | 13 | 16 | 25 | 9 | 12 |
|  |  |  |  |  |  |  |
| ***Munda*** |  |  |  |  |  |  |
| Sample 1 | 15 | 13 | 19 | 22 | 10 | 12 |
| Sample 2 | 15 | 13 | 16 | 25 | 8 | 12 |
| Sample 3 | 14 | 13 | 15 | 23 | 9 | 12 |
| Sample 4 | 14 | 12 | 17 | 22 | 9 | 12 |
| Sample 5 | 14 | 13 | 16 | 23 | 10 | 12 |
| Sample 6 | 15 | 13 | 16 | 25 | 8 | 12 |
| Sample 7 | 15 | 13 | 16 | 24 | 8 | 12 |
| Sample 8 | 15 | 13 | 16 | 24 | 8 | 12 |
| Sample 9 | 15 | 13 | 16 | 25 | 9 | 12 |
| Sample 10 | 16 | 14 | 16 | 22 | 10 | 12 |
| Sample 11 | 15 | 14 | 17 | 26 | 9 | 12 |
| Sample 12 | 13 | 14 | 17 | 22 | 9 | 12 |
| Sample 13 | 16 | 14 | 16 | 22 | 10 | 12 |
| Sample 14 | 14 | 12 | 15 | 23 | 9 | 12 |
| Sample 15 | 14 | 12 | 17 | 22 | 9 | 12 |
| Sample 16 | 15 | 14 | 17 | 22 | 9 | 12 |
| Sample 17 | 14 | 12 | 16 | 23 | 9 | 12 |
| Sample 18 | 14 | 14 | 17 | 22 | 9 | 12 |
| Sample 19 | 14 | 14 | 17 | 22 | 9 | 12 |
| Sample 20 | 16 | 14 | 16 | 22 | 9 | 12 |
| Sample 21 | 16 | 13 | 16 | 25 | 9 | 12 |
| Sample 22 | 15 | 13 | 16 | 25 | 9 | 12 |
| Sample 23 | 14 | 12 | 17 | 22 | 9 | 12 |
|  |  |  |  |  |  |  |
| ***Santhal*** |  |  |  |  |  |  |
| Sample 1 | 14 | 12 | 17 | 23 | 10 | 12 |
| Sample 2 | 15 | 14 | 17 | 22 | 9 | 12 |
| Sample 3 | 15 | 14 | 17 | 22 | 9 | 12 |
| Sample 4 | 15 | 14 | 17 | 22 | 9 | 12 |
| Sample 5 | 15 | 14 | 17 | 22 | 9 | 12 |
| Sample 6 | 14 | 14 | 16 | 24 | 8 | 12 |
| Sample 7 | 14 | 12 | 17 | 23 | 10 | 12 |
| Sample 8 | 15 | 13 | 16 | 24 | 9 | 12 |
| Sample 9 | 15 | 14 | 17 | 22 | 9 | 12 |
| Sample 10 | 16 | 14 | 17 | 25 | 10 | 12 |
| Sample 11 | 15 | 13 | 18 | 22 | 10 | 12 |
| Sample 12 | 15 | 14 | 17 | 22 | 9 | 12 |
| Sample 13 | 14 | 14 | 16 | 24 | 8 | 12 |
| Sample 14 | 16 | 13 | 16 | 24 | 9 | 12 |
| Sample 15 | 14 | 12 | 17 | 23 | 10 | 12 |
| Sample 16 | 14 | 14 | 21 | 24 | 8 | 12 |
| Sample 17 | 15 | 13 | 16 | 24 | 9 | 12 |
| Sample 18 | 16 | 14 | 21 | 22 | 9 | 12 |
| Sample 19 | 15 | 14 | 17 | 22 | 9 | 12 |
| Sample 20 | 16 | 14 | 16 | 22 | 9 | 12 |
| Sample 21 | 15 | 14 | 17 | 22 | 9 | 12 |
| Sample 22 | 15 | 14 | 16 | 21 | 10 | 12 |
| Sample 23 | 14 | 12 | 17 | 24 | 10 | 12 |
| Sample 24 | 16 | 13 | 18 | 25 | 10 | 12 |
| Sample 25 | 15 | 13 | 16 | 25 | 12 | 12 |
| Sample 26 | 15 | 13 | 16 | 25 | 9 | 12 |
| Sample 27 | 16 | 13 | 16 | 26 | 9 | 12 |
| Sample 28 | 15 | 12 | 18 | 23 | 9 | 12 |
| Sample 29 | 14 | 12 | 17 | 23 | 10 | 12 |
| Sample 30 | 15 | 14 | 17 | 22 | 9 | 12 |
| Sample 31 | 15 | 13 | 16 | 24 | 9 | 12 |
| Sample 32 | 15 | 13 | 15 | 24 | 9 | 12 |
| Sample 33 | 15 | 13 | 16 | 25 | 9 | 12 |
| Sample 34 | 16 | 14 | 16 | 22 | 9 | 12 |
| Sample 35 | 15 | 13 | 17 | 25 | 10 | 12 |
| Sample 36 | 15 | 13 | 15 | 25 | 9 | 12 |
| Sample 37 | 15 | 12 | 16 | 23 | 9 | 12 |
| Sample 38 | 16 | 12 | 16 | 23 | 9 | 12 |
|  |  |  |  |  |  |  |
| **Khasi** |  |  |  |  |  |  |
| ***Maram*** |  |  |  |  |  |  |
| Sample 1 | 15 | 9 | 17 | 28 | 10 | 16 |
| Sample 2 | 14 | 10 | 16 | 26 | 10 | 13 |
| Sample 3 | 15 | 9 | 17 | 29 | 10 | 13 |
| Sample 4 | 12 | 12 | 14 | 22 | 10 | 12 |
| Sample 5 | 14 | 11 | 15 | 26 | 10 | 13 |
| Sample 6 | 14 | 9 | 18 | 26 | 10 | 12 |
| Sample 7 | 15 | 10 | 17 | 27 | 11 | 14 |
| Sample 8 | 15 | 9 | 18 | 28 | 11 | 13 |
| Sample 9 | 14 | 9 | 18 | 27 | 11 | 13 |
| Sample 10 | 14 | 8 | 19 | 27 | 10 | 13 |
| Sample 11 | 14 | 10 | 16 | 26 | 10 | 14 |
| Sample 12 | 15 | 9 | 21 | 27 | 10 | 13 |
| Sample 13 | 17 | 9 | 19 | 28 | 10 | 14 |
| Sample 14 | 15 | 9 | 18 | 28 | 10 | 14 |
| Sample 15 | 11 | 11 | 20 | 26 | 10 | 15 |
| Sample 16 | 10 | 10 | 18 | 26 | 10 | 14 |
| Sample 17 | 15 | 11 | 16 | 26 | 11 | 13 |
| Sample 18 | 15 | 11 | 15 | 28 | 10 | 13 |
| Sample 19 | 13 | 11 | 16 | 25 | 11 | 11 |
| Sample 20 | 15 | 10 | 16 | 23 | 10 | 11 |
| Sample 21 | 12 | 12 | 16 | 22 | 10 | 11 |
| Sample 22 | 18 | 11 | 18 | 25 | 10 | 12 |
| Sample 23 | 15 | 9 | 17 | 28 | 10 | 16 |
| Sample 24 | 12 | 11 | 15 | 26 | 10 | 16 |
| Sample 25 | 14 | 10 | 16 | 26 | 10 | 13 |
| Sample 26 | 15 | 9 | 17 | 29 | 10 | 13 |
| Sample 27 | 16 | 11 | 17 | 28 | 11 | 12 |
| Sample 28 | 18 | 11 | 17 | 28 | 10 | 12 |
| Sample 29 | 15 | 11 | 18 | 29 | 13 | 13 |
| Sample 30 | 15 | 10 | 17 | 28 | 10 | 13 |
| Sample 31 | 15 | 10 | 15 | 29 | 11 | 13 |
| Sample 32 | 15 | 10 | 16 | 27 | 11 | 13 |
| Sample 33 | 15 | 11 | 15 | 26 | 10 | 14 |
| Sample 34 | 16 | 10 | 17 | 28 | 10 | 13 |
| Sample 35 | 15 | 11 | 20 | 25 | 13 | 11 |
| Sample 36 | 15 | 11 | 17 | 26 | 10 | 12 |
| Sample 37 | 15 | 10 | 18 | 26 | 10 | 12 |
| Sample 38 | 14 | 9 | 19 | 26 | 11 | 12 |
| Sample 39 | 15 | 10 | 17 | 25 | 11 | 12 |
| Sample 40 | 15 | 10 | 19 | 26 | 10 | 12 |
| Sample 41 | 15 | 11 | 17 | 28 | 10 | 12 |
| Sample 42 | 15 | 11 | 17 | 28 | 10 | 12 |
| Sample 43 | 14 | 10 | 18 | 28 | 10 | 12 |
| Sample 44 | 15 | 11 | 15 | 26 | 11 | 14 |
| Sample 45 | 15 | 10 | 17 | 27 | 10 | 14 |
| Sample 46 | 16 | 10 | 18 | 26 | 11 | 11 |
| Sample 47 | 16 | 11 | 16 | 28 | 11 | 14 |
| Sample 48 | 16 | 10 | 16 | 28 | 11 | 12 |
| Sample 49 | 15 | 11 | 20 | 26 | 11 | 12 |
| Sample 50 | 16 | 11 | 17 | 28 | 11 | 12 |
| Sample 51 | 15 | 9 | 18 | 28 | 10 | 13 |
| Sample 52 | 16 | 10 | 17 | 28 | 10 | 12 |
| Sample 53 | 14 | 10 | 19 | 27 | 11 | 12 |
| Sample 54 | 15 | 10 | 16 | 27 | 11 | 13 |
| Sample 55 | 15 | 10 | 16 | 28 | 11 | 13 |
| Sample 56 | 14 | 9 | 17 | 26 | 10 | 12 |
| Sample 57 | 15 | 11 | 15 | 26 | 11 | 11 |
| Sample 58 | 14 | 9 | 18 | 27 | 11 | 13 |
|  |  |  |  |  |  |  |
| ***Khynriam*** |  |  |  |  |  |  |
| Sample 1 | 15 | 9 | 19 | 26 | 11 | 13 |
| Sample 2 | 16 | 10 | 17 | 27 | 10 | 11 |
| Sample 3 | 15 | 10 | 17 | 26 | 11 | 12 |
| Sample 4 | 16 | 11 | 18 | 26 | 11 | 12 |
| Sample 5 | 14 | 10 | 18 | 26 | 10 | 12 |
| Sample 6 | 13 | 10 | 18 | 26 | 10 | 13 |
| Sample 7 | 15 | 9 | 19 | 26 | 10 | 15 |
| Sample 8 | 16 | 12 | 16 | 26 | 10 | 12 |
| Sample 9 | 15 | 11 | 18 | 28 | 10 | 12 |
| Sample 10 | 15 | 10 | 16 | 28 | 10 | 13 |
| Sample 11 | 15 | 11 | 18 | 26 | 10 | 12 |
| Sample 12 | 16 | 10 | 17 | 27 | 11 | 12 |
| Sample 13 | 15 | 11 | 15 | 28 | 10 | 14 |
| Sample 14 | 14 | 10 | 16 | 26 | 10 | 11 |
| Sample 15 | 15 | 10 | 16 | 26 | 11 | 15 |
| Sample 16 | 16 | 10 | 16 | 28 | 11 | 16 |
| Sample 17 | 15 | 11 | 18 | 27 | 10 | 13 |
| Sample 18 | 15 | 9 | 17 | 28 | 10 | 12 |
| Sample 19 | 16 | 11 | 15 | 28 | 10 | 15 |
| Sample 20 | 15 | 9 | 17 | 28 | 10 | 16 |
| Sample 21 | 15 | 9 | 16 | 28 | 10 | 14 |
| Sample 22 | 15 | 9 | 17 | 28 | 10 | 11 |
| Sample 23 | 14 | 9 | 17 | 26 | 10 | 13 |
| Sample 24 | 10 | 10 | 18 | 28 | 11 | 13 |
| Sample 25 | 11 | 10 | 19 | 26 | 10 | 12 |
| Sample 26 | 12 | 10 | 15 | 27 | 10 | 13 |
| Sample 27 | 12 | 10 | 18 | 26 | 10 | 14 |
| Sample 28 | 13 | 10 | 18 | 26 | 11 | 12 |
| Sample 29 | 15 | 10 | 19 | 26 | 11 | 14 |
| Sample 30 | 13 | 10 | 15 | 29 | 10 | 12 |
| Sample 31 | 14 | 12 | 19 | 26 | 10 | 12 |
| Sample 32 | 15 | 11 | 19 | 26 | 11 | 12 |
| Sample 33 | 15 | 10 | 18 | 28 | 10 | 13 |
| Sample 34 | 15 | 10 | 17 | 28 | 11 | 13 |
| Sample 35 | 16 | 10 | 17 | 28 | 10 | 13 |
| Sample 36 | 15 | 12 | 14 | 28 | 10 | 14 |
| Sample 37 | 14 | 11 | 18 | 26 | 10 | 13 |
| Sample 38 | 15 | 11 | 17 | 26 | 11 | 12 |
| Sample 39 | 17 | 10 | 17 | 28 | 10 | 11 |
| Sample 40 | 15 | 9 | 17 | 29 | 11 | 14 |
| Sample 41 | 16 | 10 | 16 | 23 | 9 | 14 |
| Sample 42 | 16 | 11 | 17 | 27 | 10 | 16 |
| Sample 43 | 17 | 11 | 14 | 31 | 11 | 13 |
| Sample 44 | 15 | 10 | 13 | 27 | 11 | 14 |
| Sample 45 | 15 | 10 | 16 | 26 | 10 | 14 |
| Sample 46 | 10 | 10 | 13 | 26 | 10 | 14 |
| Sample 47 | 15 | 9 | 19 | 26 | 10 | 12 |
| Sample 48 | 15 | 9 | 19 | 26 | 10 | 13 |
| Sample 49 | 14 | 9 | 17 | 26 | 10 | 11 |
| Sample 50 | 15 | 10 | 18 | 26 | 10 | 12 |
| Sample 51 | 15 | 10 | 16 | 28 | 10 | 13 |
| Sample 52 | 14 | 9 | 17 | 31 | 10 | 11 |
| Sample 53 | 12 | 8 | 15 | 25 | 8 | 11 |
| Sample 54 | 15 | 11 | 18 | 26 | 11 | 12 |
| Sample 55 | 15 | 10 | 17 | 28 | 10 | 13 |
| Sample 56 | 16 | 10 | 16 | 27 | 10 | 13 |
| Sample 57 | 15 | 10 | 16 | 26 | 10 | 14 |
| Sample 58 | 15 | 10 | 17 | 27 | 10 | 13 |
| Sample 59 | 11 | 10 | 18 | 27 | 11 | 13 |
| Sample 60 | 15 | 10 | 18 | 26 | 10 | 13 |
| Sample 61 | 15 | 10 | 18 | 27 | 10 | 13 |
| Sample 62 | 15 | 11 | 17 | 25 | 10 | 11 |
| Sample 63 | 14 | 10 | 17 | 25 | 10 | 13 |
| Sample 64 | 15 | 9 | 18 | 26 | 10 | 11 |
| Sample 65 | 16 | 9 | 17 | 26 | 10 | 14 |
| Sample 66 | 15 | 11 | 15 | 28 | 10 | 13 |
| Sample 67 | 15 | 10 | 17 | 26 | 10 | 13 |
| Sample 68 | 15 | 10 | 17 | 24 | 10 | 13 |
| Sample 69 | 14 | 9 | 18 | 26 | 11 | 13 |
| Sample 70 | 15 | 10 | 19 | 26 | 10 | 12 |
| Sample 71 | 15 | 11 | 15 | 28 | 11 | 14 |
| Sample 72 | 14 | 9 | 18 | 27 | 10 | 13 |
| Sample 73 | 17 | 10 | 17 | 27 | 10 | 11 |
| Sample 74 | 15 | 10 | 16 | 28 | 11 | 14 |
| Sample 75 | 16 | 11 | 16 | 28 | 11 | 13 |
| Sample 76 | 16 | 10 | 17 | 28 | 11 | 13 |
| Sample 77 | 14 | 10 | 18 | 28 | 11 | 14 |
| Sample 78 | 15 | 10 | 16 | 28 | 10 | 14 |
| Sample 79 | 14 | 10 | 17 | 26 | 10 | 13 |
| Sample 80 | 14 | 9 | 16 | 27 | 10 | 11 |
| Sample 81 | 15 | 10 | 16 | 25 | 10 | 11 |
| Sample 82 | 15 | 10 | 18 | 26 | 10 | 12 |
|  |  |  |  |  |  |  |
| ***Pnar*** |  |  |  |  |  |  |
| Sample 1 | 15 | 10 | 17 | 24 | 10 | 11 |
| Sample 2 | 15 | 11 | 18 | 26 | 11 | 12 |
| Sample 3 | 16 | 11 | 20 | 28 | 11 | 12 |
| Sample 4 | 15 | 10 | 17 | 24 | 10 | 11 |
| Sample 5 | 15 | 11 | 17 | 26 | 11 | 11 |
| Sample 6 | 14 | 12 | 17 | 25 | 11 | 12 |
| Sample 7 | 15 | 11 | 17 | 26 | 11 | 16 |
| Sample 8 | 15 | 10 | 18 | 27 | 10 | 13 |
| Sample 9 | 15 | 9 | 18 | 27 | 10 | 13 |
| Sample 10 | 15 | 14 | 13 | 24 | 10 | 12 |
| Sample 11 | 16 | 10 | 17 | 23 | 10 | 11 |
| Sample 12 | 15 | 10 | 18 | 27 | 10 | 13 |
| Sample 13 | 14 | 11 | 17 | 28 | 11 | 14 |
| Sample 14 | 15 | 10 | 17 | 24 | 10 | 11 |
| Sample 15 | 15 | 12 | 16 | 26 | 10 | 11 |
| Sample 16 | 15 | 10 | 18 | 26 | 11 | 13 |
| Sample 17 | 10 | 9 | 17 | 26 | 10 | 11 |
| Sample 18 | 14 | 11 | 15 | 27 | 10 | 11 |
| Sample 19 | 14 | 9 | 17 | 26 | 10 | 12 |
| Sample 20 | 16 | 10 | 16 | 28 | 10 | 12 |
| Sample 21 | 14 | 11 | 18 | 28 | 10 | 12 |
| Sample 22 | 15 | 9 | 17 | 28 | 10 | 11 |
| Sample 23 | 15 | 9 | 17 | 28 | 10 | 11 |
| Sample 24 | 15 | 11 | 15 | 29 | 10 | 11 |
| Sample 25 | 16 | 10 | 18 | 28 | 10 | 12 |
| Sample 26 | 16 | 9 | 19 | 26 | 10 | 12 |
| Sample 27 | 15 | 10 | 18 | 26 | 10 | 12 |
| Sample 28 | 15 | 10 | 18 | 26 | 10 | 12 |
| Sample 29 | 15 | 10 | 16 | 29 | 10 | 12 |
| Sample 30 | 15 | 11 | 16 | 26 | 12 | 12 |
| Sample 31 | 15 | 11 | 17 | 26 | 10 | 12 |
| Sample 32 | 13 | 9 | 18 | 26 | 10 | 10 |
| Sample 33 | 14 | 10 | 19 | 27 | 11 | 11 |
| Sample 34 | 14 | 11 | 17 | 25 | 11 | 12 |
| Sample 35 | 14 | 11 | 15 | 25 | 11 | 12 |
| Sample 36 | 15 | 10 | 15 | 28 | 10 | 14 |
| Sample 37 | 15 | 10 | 16 | 26 | 10 | 13 |
| Sample 38 | 15 | 10 | 16 | 26 | 10 | 14 |
| Sample 39 | 15 | 10 | 16 | 28 | 10 | 13 |
| Sample 40 | 15 | 12 | 18 | 28 | 10 | 13 |
|  |  |  |  |  |  |  |
| ***Bhoi*** |  |  |  |  |  |  |
| Sample 1 | 15 | 9 | 19 | 26 | 10 | 12 |
| Sample 2 | 14 | 10 | 16 | 26 | 10 | 13 |
| Sample 3 | 15 | 10 | 17 | 26 | 11 | 12 |
| Sample 4 | 14 | 9 | 17 | 27 | 10 | 11 |
| Sample 5 | 15 | 11 | 16 | 28 | 10 | 13 |
| Sample 6 | 15 | 9 | 17 | 25 | 10 | 12 |
| Sample 7 | 16 | 9 | 17 | 25 | 10 | 12 |
| Sample 8 | 15 | 10 | 18 | 26 | 10 | 12 |
| Sample 9 | 15 | 11 | 17 | 25 | 10 | 11 |
| Sample 10 | 14 | 10 | 18 | 26 | 10 | 12 |
| Sample 11 | 15 | 9 | 17 | 26 | 10 | 11 |
| Sample 12 | 14 | 9 | 18 | 26 | 11 | 11 |
| Sample 13 | 15 | 10 | 17 | 28 | 11 | 15 |
| Sample 14 | 15 | 9 | 18 | 26 | 11 | 12 |
| Sample 15 | 11 | 9 | 19 | 26 | 11 | 11 |
| Sample 16 | 15 | 11 | 18 | 28 | 11 | 13 |
| Sample 17 | 15 | 10 | 17 | 28 | 10 | 13 |
| Sample 18 | 14 | 11 | 19 | 25 | 10 | 11 |
| Sample 19 | 15 | 10 | 17 | 26 | 11 | 13 |
| Sample 20 | 14 | 10 | 19 | 24 | 10 | 13 |
| Sample 21 | 11 | 10 | 17 | 26 | 10 | 11 |
| Sample 22 | 14 | 11 | 17 | 26 | 10 | 11 |
| Sample 23 | 17 | 9 | 22 | 26 | 10 | 13 |
| Sample 24 | 15 | 10 | 17 | 27 | 10 | 13 |
| Sample 25 | 15 | 11 | 16 | 28 | 11 | 13 |
| Sample 26 | 15 | 9 | 19 | 26 | 10 | 11 |
| Sample 27 | 11 | 11 | 16 | 28 | 11 | 11 |
| Sample 28 | 14 | 10 | 16 | 28 | 10 | 13 |
| Sample 29 | 15 | 10 | 18 | 28 | 10 | 13 |
| Sample 30 | 14 | 11 | 17 | 27 | 10 | 11 |
|  |  |  |  |  |  |  |
| ***War Khasi*** |  |  |  |  |  |  |
| Sample 1 | 15 | 10 | 17 | 28 | 11 | 12 |
| Sample 2 | 15 | 10 | 17 | 29 | 10 | 13 |
| Sample 3 | 15 | 9 | 19 | 27 | 10 | 13 |
| Sample 4 | 15 | 10 | 16 | 26 | 10 | 14 |
| Sample 5 | 15 | 9 | 17 | 26 | 10 | 14 |
| Sample 6 | 15 | 10 | 16 | 26 | 10 | 14 |
| Sample 7 | 16 | 11 | 15 | 29 | 11 | 13 |
| Sample 8 | 15 | 11 | 19 | 26 | 11 | 12 |
| Sample 9 | 15 | 11 | 15 | 28 | 11 | 13 |
| Sample 10 | 15 | 10 | 15 | 28 | 9 | 14 |
| Sample 11 | 15 | 11 | 17 | 26 | 11 | 12 |
| Sample 12 | 15 | 10 | 16 | 26 | 10 | 12 |
| Sample 13 | 15 | 10 | 16 | 28 | 10 | 13 |
| Sample 14 | 15 | 10 | 16 | 28 | 11 | 13 |
| Sample 15 | 15 | 10 | 15 | 26 | 10 | 14 |
| Sample 16 | 15 | 9 | 17 | 26 | 10 | 14 |
| Sample 17 | 15 | 10 | 13 | 26 | 10 | 12 |
| Sample 18 | 16 | 11 | 18 | 28 | 11 | 12 |
| Sample 19 | 14 | 10 | 18 | 29 | 10 | 12 |
| Sample 20 | 15 | 10 | 20 | 28 | 11 | 12 |
| Sample 21 | 15 | 10 | 18 | 26 | 11 | 12 |
| Sample 22 | 15 | 11 | 17 | 28 | 10 | 13 |
| Sample 23 | 16 | 9 | 18 | 26 | 10 | 11 |
|  |  |  |  |  |  |  |
| **Dravidian** |  |  |  |  |  |  |
| ***Akhutota*** |  |  |  |  |  |  |
| Sample 1 | 17 | 11 | 18 | 26 | 11 | 13 |
| Sample 2 | 15 | 12 | 16 | 23 | 10 | 14 |
| Sample 3 | 17 | 11 | 18 | 26 | 10 | 12 |
| Sample 4 | 15 | 11 | 16 | 23 | 10 | 12 |
| Sample 5 | 17 | 11 | 18 | 26 | 11 | 13 |
| Sample 6 | 15 | 10 | 16 | 25 | 10 | 13 |
| Sample 7 | 15 | 10 | 17 | 24 | 10 | 12 |
| Sample 8 | 15 | 11 | 18 | 24 | 10 | 14 |
| Sample 9 | 16 | 11 | 15 | 23 | 10 | 12 |
| Sample 10 | 16 | 11 | 16 | 26 | 11 | 13 |
| Sample 11 | 15 | 11 | 16 | 24 | 11 | 12 |
| Sample 12 | 16 | 11 | 15 | 23 | 10 | 12 |
| Sample 13 | 16 | 11 | 17 | 26 | 11 | 13 |
| Sample 14 | 16 | 11 | 16 | 22 | 11 | 12 |
| Sample 15 | 14 | 10 | 16 | 23 | 11 | 11 |
| Sample 16 | 15 | 12 | 16 | 24 | 10 | 14 |
| Sample 17 | 14 | 11 | 17 | 23 | 9 | 13 |
| Sample 18 | 14 | 12 | 15 | 22 | 10 | 14 |
| Sample 19 | 15 | 10 | 17 | 24 | 10 | 12 |
| Sample 20 | 14 | 11 | 15 | 23 | 11 | 12 |
| Sample 21 | 14 | 10 | 15 | 24 | 10 | 12 |
|  |  |  |  |  |  |  |
| ***Kapu*** |  |  |  |  |  |  |
| Sample 1 | 16 | 12 | 17 | 24 | 10 | 12 |
| Sample 2 | 15 | 12 | 17 | 25 | 11 | 13 |
| Sample 3 | 16 | 12 | 16 | 23 | 10 | 12 |
| Sample 4 | 14 | 12 | 17 | 23 | 10 | 14 |
| Sample 5 | 15 | 12 | 16 | 23 | 10 | 12 |
| Sample 6 | 15 | 11 | 18 | 20 | 10 | 13 |
| Sample 7 | 16 | 11 | 16 | 22 | 10 | 12 |
| Sample 8 | 15 | 11 | 18 | 25 | 11 | 12 |
| Sample 9 | 14 | 10 | 17 | 22 | 10 | 10 |
| Sample 10 | 16 | 10 | 17 | 23 | 10 | 12 |
| Sample 11 | 15 | 11 | 18 | 25 | 11 | 12 |
| Sample 12 | 14 | 11 | 15 | 24 | 10 | 14 |
| Sample 13 | 15 | 12 | 16 | 22 | 10 | 11 |
| Sample 14 | 14 | 10 | 17 | 22 | 10 | 10 |
| Sample 15 | 14 | 10 | 16 | 22 | 10 | 10 |
| Sample 16 | 15 | 10 | 16 | 22 | 10 | 10 |
|  |  |  |  |  |  |  |
| ***Panta*** |  |  |  |  |  |  |
| Sample 1 | 16 | 11 | 17 | 26 | 11 | 13 |
| Sample 2 | 17 | 10 | 17 | 24 | 10 | 11 |
| Sample 3 | 17 | 11 | 17 | 24 | 10 | 12 |
| Sample 4 | 16 | 11 | 18 | 23 | 10 | 12 |
| Sample 5 | 14 | 11 | 18 | 24 | 10 | 14 |
| Sample 6 | 14 | 10 | 16 | 23 | 10 | 11 |
| Sample 7 | 14 | 9 | 17 | 23 | 10 | 13 |
| Sample 8 | 15 | 12 | 17 | 21 | 10 | 14 |
| Sample 9 | 15 | 10 | 16 | 24 | 11 | 12 |
| Sample 10 | 15 | 12 | 17 | 23 | 10 | 12 |
| Sample 11 | 16 | 10 | 19 | 26 | 10 | 13 |
| Sample 12 | 15 | 10 | 15 | 24 | 10 | 12 |
| Sample 13 | 14 | 12 | 16 | 23 | 10 | 13 |
| Sample 14 | 15 | 12 | 17 | 21 | 10 | 14 |
| Sample 15 | 15 | 12 | 17 | 23 | 10 | 13 |
| Sample 16 | 16 | 12 | 17 | 22 | 10 | 12 |
| Sample 17 | 15 | 11 | 19 | 22 | 10 | 12 |
| Sample 18 | 15 | 11 | 19 | 21 | 10 | 12 |
| Sample 19 | 16 | 12 | 18 | 23 | 10 | 13 |
| Sample 20 | 17 | 11 | 16 | 24 | 11 | 12 |
| Sample 21 | 14 | 11 | 17 | 24 | 10 | 13 |
|  |  |  |  |  |  |  |
| ***Pokanati*** |  |  |  |  |  |  |
| Sample 1 | 16 | 11 | 16 | 23 | 10 | 12 |
| Sample 2 | 14 | 11 | 16 | 24 | 10 | 13 |
| Sample 3 | 16 | 11 | 17 | 22 | 10 | 12 |
| Sample 4 | 15 | 10 | 15 | 23 | 11 | 13 |
| Sample 5 | 18 | 10 | 15 | 26 | 11 | 13 |
| Sample 6 | 14 | 12 | 16 | 23 | 10 | 15 |
| Sample 7 | 16 | 10 | 16 | 23 | 10 | 12 |
| Sample 8 | 16 | 10 | 16 | 24 | 10 | 12 |
| Sample 9 | 16 | 11 | 17 | 25 | 10 | 14 |
| Sample 10 | 16 | 12 | 17 | 26 | 11 | 12 |
| Sample 11 | 15 | 11 | 17 | 25 | 10 | 14 |
| Sample 12 | 14 | 11 | 17 | 24 | 10 | 14 |
| Sample 13 | 14 | 11 | 17 | 24 | 11 | 12 |
| Sample 14 | 15 | 12 | 17 | 24 | 11 | 13 |
| Sample 15 | 16 | 11 | 18 | 26 | 12 | 13 |
| Sample 16 | 15 | 12 | 17 | 24 | 11 | 13 |
| Sample 17 | 15 | 12 | 16 | 24 | 11 | 14 |
| Sample 18 | 15 | 11 | 18 | 22 | 11 | 12 |
| Sample 19 | 16 | 11 | 17 | 22 | 11 | 12 |
| Sample 20 | 17 | 12 | 17 | 23 | 11 | 11 |
| Sample 21 | 17 | 12 | 16 | 22 | 11 | 13 |
| Sample 22 | 17 | 11 | 17 | 26 | 11 | 13 |
| Sample 23 | 16 | 12 | 16 | 22 | 11 | 12 |
| Sample 24 | 15 | 10 | 16 | 22 | 11 | 11 |
| Sample 25 | 16 | 12 | 17 | 24 | 11 | 12 |
|  |  |  |  |  |  |  |
| ***Vanne*** |  |  |  |  |  |  |
| Sample 1 | 16 | 11 | 16 | 24 | 10 | 14 |
| Sample 2 | 15 | 13 | 16 | 21 | 10 | 13 |
| Sample 3 | 15 | 12 | 17 | 25 | 10 | 13 |
| Sample 4 | 14 | 11 | 21 | 23 | 10 | 12 |
| Sample 5 | 14 | 10 | 16 | 22 | 10 | 11 |
| Sample 6 | 15 | 11 | 18 | 26 | 11 | 11 |
| Sample 7 | 15 | 11 | 16 | 24 | 10 | 13 |
| Sample 8 | 14 | 10 | 17 | 22 | 10 | 10 |
| Sample 9 | 14 | 11 | 19 | 23 | 10 | 12 |
| Sample 10 | 15 | 10 | 17 | 23 | 10 | 11 |
| Sample 11 | 14 | 11 | 17 | 24 | 10 | 13 |
| Sample 12 | 14 | 11 | 17 | 23 | 10 | 11 |
| Sample 13 | 15 | 12 | 17 | 22 | 10 | 12 |
| Sample 14 | 14 | 10 | 16 | 22 | 10 | 11 |
| Sample 15 | 16 | 12 | 16 | 23 | 11 | 12 |
| Sample 16 | 15 | 11 | 16 | 24 | 10 | 13 |
| Sample 17 | 17 | 11 | 18 | 25 | 11 | 12 |
| Sample 18 | 15 | 11 | 16 | 24 | 11 | 14 |
| Sample 19 | 15 | 11 | 17 | 23 | 11 | 12 |
| Sample 20 | 14 | 11 | 16 | 24 | 10 | 13 |
| Sample 21 | 15 | 11 | 16 | 25 | 10 | 13 |
| Sample 22 | 15 | 11 | 15 | 23 | 11 | 12 |
| Sample 23 | 15 | 12 | 17 | 22 | 10 | 12 |
