# Supplementary material for: Global Patterns in Human Mitochondrial DNA and Y-Chromosome Variation Caused by Spatial Instability of the Local Cultural Processes
Source: PLoS Genet. 2006 Apr 14;2(4):e53. doi: 10.1371/journal.pgen.0020053 (PMC1435684; doi:10.1371/journal.pgen.0020053)
Supplement: Supplementary file 2 [file pgen.0020053.st002.doc]

Supplementray Table 2: Y-chromosome Haplotypes based on 9 Y-STRs for 6 Populations

|  | **388** | **426** | **437** | **438** | **439** | **447** | **448** | **460** | **H4** |
| --- | --- | --- | --- | --- | --- | --- | --- | --- | --- |
| **Mundari** |  |  |  |  |  |  |  |  |  |
| ***Bhumij*** |  |  |  |  |  |  |  |  |  |
| Sample 1 | 12 | 11 | 14 | 10 | 17 | 27 | 20 | 9 | 11 |
| Sample 2 | 12 | 11 | 14 | 10 | 19 | 27 | 21 | 9 | 11 |
| Sample 3 | 12 | 11 | 14 | 10 | 17 | 27 | 20 | 9 | 11 |
| Sample 4 | 12 | 11 | 14 | 10 | 17 | 27 | 20 | 9 | 11 |
| Sample 5 | 12 | 11 | 14 | 10 | 19 | 27 | 21 | 9 | 11 |
| Sample 6 | 12 | 11 | 14 | 10 | 19 | 27 | 21 | 9 | 11 |
| Sample 7 | 12 | 11 | 14 | 10 | 19 | 27 | 21 | 9 | 11 |
| Sample 8 | 12 | 11 | 14 | 10 | 17 | 27 | 21 | 9 | 11 |
| Sample 9 | 9 | 11 | 14 | 11 | 17 | 23 | 24 | 9 | 11 |
| Sample 10 | 12 | 11 | 14 | 11 | 18 | 27 | 20 | 9 | 11 |
| Sample 11 | 12 | 11 | 14 | 10 | 17 | 27 | 20 | 9 | 11 |
| Sample 12 | 12 | 11 | 14 | 9 | 16 | 23 | 22 | 9 | 13 |
| Sample 13 | 12 | 11 | 14 | 11 | 18 | 27 | 20 | 9 | 11 |
| Sample 14 | 12 | 12 | 14 | 11 | 15 | 24 | 23 | 10 | 11 |
| Sample 15 | 9 | 11 | 14 | 11 | 17 | 23 | 24 | 9 | 11 |
| Sample 16 | 12 | 11 | 14 | 9 | 16 | 23 | 22 | 9 | 13 |
| Sample 17 | 11 | 11 | 14 | 10 | 18 | 23 | 21 | 9 | 9 |
| Sample 18 | 9 | 11 | 14 | 11 | 17 | 23 | 24 | 9 | 11 |
| Sample 19 | 12 | 11 | 14 | 10 | 18 | 27 | 21 | 9 | 11 |
| Sample 20 | 12 | 11 | 14 | 9 | 16 | 22 | 22 | 9 | 13 |
| Sample 21 | 12 | 11 | 14 | 10 | 17 | 27 | 21 | 9 | 11 |
| Sample 22 | 12 | 11 | 14 | 10 | 18 | 27 | 21 | 9 | 11 |
| Sample 23 | 12 | 11 | 14 | 10 | 17 | 27 | 20 | 9 | 11 |
| Sample 24 | 12 | 11 | 14 | 10 | 17 | 27 | 21 | 9 | 11 |
| Sample 25 | 12 | 11 | 14 | 9 | 16 | 22 | 22 | 9 | 12 |
| Sample 26 | 12 | 12 | 14 | 11 | 15 | 24 | 23 | 11 | 11 |
| Sample 27 | 12 | 11 | 14 | 9 | 16 | 23 | 22 | 9 | 13 |
| Sample 28 | 12 | 11 | 14 | 9 | 16 | 23 | 22 | 9 | 13 |
| Sample 29 | 12 | 11 | 14 | 10 | 18 | 27 | 21 | 9 | 11 |
| Sample 30 | 12 | 11 | 14 | 10 | 18 | 27 | 20 | 9 | 11 |
| Sample 31 | 12 | 12 | 16 | 11 | 15 | 27 | 22 | 10 | 12 |
| Sample 32 | 12 | 11 | 14 | 10 | 18 | 25 | 21 | 9 | 11 |
| Sample 33 | 12 | 11 | 14 | 10 | 19 | 27 | 21 | 9 | 11 |
| Sample 34 | 12 | 12 | 16 | 11 | 15 | 27 | 22 | 10 | 12 |
| Sample 35 | 12 | 11 | 14 | 9 | 16 | 23 | 22 | 9 | 13 |
| Sample 36 | 12 | 11 | 14 | 10 | 17 | 22 | 21 | 10 | 13 |
| Sample 37 | 12 | 11 | 14 | 10 | 17 | 27 | 21 | 9 | 11 |
| Sample 38 | 12 | 11 | 14 | 10 | 17 | 27 | 21 | 9 | 11 |
| Sample 39 | 12 | 11 | 14 | 10 | 17 | 26 | 22 | 10 | 13 |
|  |  |  |  |  |  |  |  |  |  |
| ***Munda*** |  |  |  |  |  |  |  |  |  |
| Sample 1 | 11 | 12 | 14 | 11 | 15 | 23 | 23 | 10 | 13 |
| Sample 2 | 12 | 11 | 14 | 10 | 17 | 27 | 21 | 9 | 11 |
| Sample 3 | 12 | 11 | 15 | 11 | 16 | 24 | 22 | 9 | 11 |
| Sample 4 | 12 | 11 | 14 | 9 | 16 | 23 | 23 | 9 | 13 |
| Sample 5 | 12 | 12 | 15 | 12 | 18 | 25 | 22 | 9 | 12 |
| Sample 6 | 12 | 11 | 14 | 10 | 17 | 27 | 21 | 9 | 11 |
| Sample 7 | 12 | 11 | 14 | 10 | 17 | 27 | 21 | 9 | 11 |
| Sample 8 | 12 | 11 | 14 | 10 | 17 | 27 | 21 | 9 | 11 |
| Sample 9 | 12 | 11 | 14 | 11 | 18 | 27 | 21 | 9 | 11 |
| Sample 10 | 12 | 11 | 14 | 9 | 15 | 23 | 22 | 10 | 13 |
| Sample 11 | 12 | 12 | 15 | 11 | 15 | 25 | 23 | 10 | 12 |
| Sample 12 | 8 | 12 | 14 | 11 | 17 | 26 | 22 | 8 | 10 |
| Sample 13 | 12 | 11 | 14 | 9 | 15 | 23 | 22 | 10 | 13 |
| Sample 14 | 13 | 12 | 16 | 11 | 15 | 26 | 23 | 10 | 12 |
| Sample 15 | 12 | 11 | 14 | 9 | 16 | 23 | 23 | 9 | 12 |
| Sample 16 | 13 | 12 | 16 | 11 | 15 | 26 | 23 | 10 | 12 |
| Sample 17 | 13 | 12 | 16 | 11 | 15 | 26 | 23 | 10 | 12 |
| Sample 18 | 13 | 12 | 16 | 11 | 15 | 26 | 23 | 10 | 12 |
| Sample 19 | 13 | 12 | 16 | 11 | 15 | 26 | 23 | 10 | 12 |
| Sample 20 | 12 | 11 | 14 | 9 | 16 | 23 | 22 | 9 | 12 |
| Sample 21 | 12 | 11 | 14 | 10 | 17 | 27 | 21 | 9 | 11 |
| Sample 22 | 12 | 11 | 14 | 10 | 20 | 28 | 21 | 9 | 11 |
| Sample 23 | 12 | 11 | 14 | 9 | 16 | 23 | 23 | 9 | 12 |
|  |  |  |  |  |  |  |  |  |  |
| ***Santhal*** |  |  |  |  |  |  |  |  |  |
| Sample 1 | 12 | 11 | 14 | 10 | 17 | 22 | 22 | 10 | 12 |
| Sample 2 | 12 | 11 | 14 | 9 | 16 | 23 | 22 | 9 | 13 |
| Sample 3 | 12 | 11 | 14 | 9 | 16 | 23 | 22 | 9 | 13 |
| Sample 4 | 12 | 11 | 14 | 10 | 16 | 23 | 22 | 9 | 13 |
| Sample 5 | 12 | 11 | 14 | 10 | 16 | 23 | 22 | 9 | 13 |
| Sample 6 | 12 | 12 | 16 | 11 | 18 | 26 | 23 | 9 | 13 |
| Sample 7 | 12 | 11 | 14 | 10 | 17 | 22 | 22 | 10 | 12 |
| Sample 8 | 12 | 11 | 14 | 10 | 17 | 27 | 21 | 10 | 11 |
| Sample 9 | 12 | 11 | 14 | 9 | 16 | 23 | 22 | 9 | 13 |
| Sample 10 | 12 | 11 | 14 | 10 | 17 | 28 | 20 | 9 | 10 |
| Sample 11 | 13 | 11 | 14 | 10 | 17 | 22 | 21 | 8 | 12 |
| Sample 12 | 12 | 11 | 14 | 10 | 16 | 23 | 22 | 9 | 13 |
| Sample 13 | 12 | 12 | 16 | 11 | 18 | 26 | 23 | 9 | 13 |
| Sample 14 | 12 | 11 | 14 | 10 | 17 | 27 | 21 | 9 | 11 |
| Sample 15 | 12 | 11 | 14 | 10 | 17 | 22 | 22 | 10 | 13 |
| Sample 16 | 12 | 12 | 16 | 11 | 18 | 26 | 23 | 9 | 13 |
| Sample 17 | 12 | 11 | 14 | 12 | 17 | 26 | 21 | 9 | 11 |
| Sample 18 | 12 | 11 | 14 | 9 | 16 | 23 | 22 | 9 | 13 |
| Sample 19 | 12 | 11 | 14 | 9 | 16 | 23 | 22 | 9 | 12 |
| Sample 20 | 12 | 11 | 14 | 9 | 16 | 23 | 22 | 9 | 13 |
| Sample 21 | 12 | 11 | 14 | 9 | 16 | 23 | 22 | 9 | 13 |
| Sample 22 | 12 | 11 | 15 | 10 | 17 | 23 | 22 | 8 | 11 |
| Sample 23 | 12 | 11 | 14 | 10 | 17 | 22 | 22 | 10 | 12 |
| Sample 24 | 12 | 12 | 14 | 11 | 15 | 24 | 23 | 10 | 13 |
| Sample 25 | 12 | 11 | 14 | 11 | 17 | 27 | 21 | 9 | 11 |
| Sample 26 | 12 | 11 | 14 | 10 | 17 | 28 | 21 | 9 | 11 |
| Sample 27 | 13 | 11 | 14 | 11 | 18 | 25 | 22 | 9 | 12 |
| Sample 29 | 12 | 11 | 14 | 10 | 17 | 22 | 22 | 10 | 12 |
| Sample 30 | 12 | 11 | 14 | 9 | 16 | 23 | 22 | 9 | 13 |
| Sample 31 | 12 | 11 | 14 | 10 | 17 | 27 | 21 | 9 | 11 |
| Sample 32 | 12 | 11 | 14 | 10 | 18 | 27 | 21 | 9 | 11 |
| Sample 33 | 11 | 11 | 14 | 10 | 17 | 26 | 20 | 9 | 10 |
| Sample 34 | 12 | 11 | 14 | 9 | 16 | 24 | 22 | 9 | 13 |
| Sample 35 | 12 | 11 | 14 | 10 | 17 | 26 | 21 | 9 | 11 |
| Sample 36 | 12 | 11 | 14 | 10 | 17 | 25 | 21 | 9 | 11 |
| Sample 37 | 9 | 11 | 14 | 11 | 16 | 23 | 23 | 9 | 11 |
| Sample 38 | 9 | 11 | 14 | 11 | 16 | 23 | 23 | 9 | 11 |
|  |  |  |  |  |  |  |  |  |  |
| **Khasi** |  |  |  |  |  |  |  |  |  |
| ***Maram*** |  |  |  |  |  |  |  |  |  |
| Sample 1 | 14 | 10 | 14 | 12 | 19 | 23 | 23 | 10 | 11 |
| Sample 2 | 14 | 12 | 14 | 13 | 17 | 26 | 24 | 10 | 12 |
| Sample 3 | 14 | 11 | 14 | 11 | 18 | 28 | 22 | 9 | 11 |
| Sample 4 | 14 | 11 | 14 | 11 | 18 | 28 | 22 | 10 | 10 |
| Sample 5 | 15 | 10 | 15 | 12 | 19 | 27 | 25 | 10 | 10 |
| Sample 6 | 14 | 10 | 15 | 11 | 18 | 25 | 23 | 8 | 10 |
| Sample 7 | 14 | 11 | 14 | 11 | 18 | 27 | 22 | 9 | 11 |
| Sample 8 | 14 | 10 | 14 | 12 | 18 | 25 | 23 | 9 | 11 |
| Sample 9 | 14 | 10 | 15 | 12 | 19 | 27 | 22 | 10 | 10 |
| Sample 10 | 14 | 11 | 14 | 12 | 19 | 27 | 22 | 9 | 11 |
| Sample 11 | 14 | 12 | 14 | 12 | 19 | 28 | 22 | 9 | 11 |
| Sample 12 | 12 | 11 | 15 | 13 | 18 | 24 | 24 | 8 | 13 |
| Sample 13 | 14 | 11 | 14 | 12 | 20 | 28 | 22 | 9 | 10 |
| Sample 14 | 14 | 12 | 14 | 10 | 18 | 24 | 23 | 10 | 10 |
| Sample 15 | 14 | 10 | 14 | 11 | 20 | 28 | 22 | 9 | 11 |
| Sample 16 | 12 | 10 | 15 | 13 | 20 | 24 | 24 | 9 | 11 |
| Sample 17 | 14 | 10 | 14 | 12 | 18 | 28 | 22 | 10 | 11 |
| Sample 18 | 14 | 11 | 14 | 11 | 19 | 28 | 22 | 9 | 11 |
| Sample 19 | 14 | 11 | 14 | 9 | 18 | 25 | 23 | 9 | 10 |
| Sample 20 | 14 | 10 | 15 | 11 | 19 | 24 | 24 | 10 | 12 |
| Sample 21 | 14 | 10 | 14 | 12 | 19 | 28 | 22 | 9 | 10 |
| Sample 22 | 15 | 11 | 14 | 11 | 19 | 27 | 25 | 10 | 9 |
| Sample 23 | 14 | 11 | 14 | 11 | 20 | 26 | 22 | 9 | 11 |
| Sample 24 | 15 | 11 | 14 | 12 | 19 | 27 | 25 | 10 | 9 |
| Sample 25 | 14 | 11 | 14 | 12 | 18 | 26 | 23 | 8 | 10 |
| Sample 26 | 14 | 10 | 14 | 11 | 19 | 28 | 22 | 9 | 10 |
| Sample 27 | 14 | 10 | 14 | 12 | 19 | 28 | 22 | 9 | 10 |
| Sample 28 | 14 | 10 | 16 | 13 | 17 | 28 | 23 | 9 | 11 |
| Sample 29 | 14 | 11 | 14 | 13 | 18 | 28 | 22 | 10 | 12 |
| Sample 30 | 14 | 11 | 14 | 12 | 18 | 28 | 22 | 10 | 11 |
| Sample 31 | 14 | 11 | 14 | 12 | 19 | 28 | 22 | 9 | 11 |
|  |  |  |  |  |  |  |  |  |  |
| ***Khynriam*** |  |  |  |  |  |  |  |  |  |
| Sample 1 | 14 | 11 | 14 | 12 | 19 | 26 | 22 | 9 | 11 |
| Sample 2 | 14 | 11 | 14 | 12 | 20 | 27 | 22 | 10 | 9 |
| Sample 3 | 15 | 11 | 14 | 12 | 19 | 27 | 25 | 10 | 10 |
| Sample 4 | 14 | 11 | 14 | 11 | 19 | 28 | 22 | 9 | 11 |
| Sample 5 | 14 | 10 | 14 | 11 | 20 | 27 | 22 | 10 | 9 |
| Sample 6 | 16 | 11 | 15 | 12 | 18 | 26 | 24 | 10 | 12 |
| Sample 7 | 14 | 11 | 14 | 12 | 19 | 27 | 22 | 9 | 11 |
| Sample 8 | 14 | 12 | 14 | 14 | 17 | 26 | 25 | 9 | 13 |
| Sample 9 | 14 | 11 | 14 | 12 | 19 | 28 | 22 | 9 | 11 |
| Sample 10 | 14 | 11 | 15 | 13 | 19 | 24 | 24 | 9 | 12 |
| Sample 11 | 14 | 11 | 14 | 12 | 19 | 27 | 22 | 9 | 11 |
| Sample 12 | 12 | 11 | 16 | 13 | 19 | 24 | 24 | 8 | 12 |
| Sample 13 | 15 | 11 | 14 | 11 | 20 | 27 | 25 | 10 | 9 |
| Sample 14 | 14 | 11 | 15 | 12 | 18 | 25 | 23 | 8 | 10 |
| Sample 15 | 14 | 11 | 15 | 12 | 19 | 25 | 23 | 8 | 10 |
| Sample 16 | 14 | 11 | 14 | 12 | 18 | 25 | 23 | 8 | 10 |
| Sample 17 | 14 | 12 | 14 | 13 | 17 | 27 | 24 | 10 | 11 |
| Sample 18 | 14 | 11 | 14 | 12 | 20 | 27 | 22 | 9 | 11 |
| Sample 19 | 15 | 12 | 15 | 14 | 18 | 27 | 23 | 9 | 11 |
| Sample 20 | 14 | 11 | 14 | 11 | 19 | 28 | 22 | 8 | 12 |
| Sample 21 | 14 | 12 | 14 | 13 | 17 | 25 | 24 | 11 | 12 |
| Sample 22 | 14 | 11 | 16 | 12 | 18 | 25 | 22 | 8 | 10 |
| Sample 23 | 15 | 11 | 14 | 12 | 19 | 27 | 25 | 10 | 10 |
| Sample 24 | 14 | 11 | 14 | 12 | 19 | 28 | 22 | 9 | 11 |
| Sample 25 | 14 | 11 | 14 | 12 | 18 | 27 | 22 | 9 | 11 |
| Sample 26 | 19 | 11 | 14 | 10 | 18 | 27 | 24 | 10 | 10 |
| Sample 27 | 14 | 11 | 15 | 12 | 18 | 25 | 23 | 8 | 10 |
| Sample 28 | 14 | 11 | 15 | 12 | 18 | 25 | 24 | 8 | 11 |
| Sample 29 | 14 | 11 | 14 | 12 | 18 | 27 | 22 | 10 | 9 |
| Sample 30 | 14 | 11 | 14 | 11 | 19 | 27 | 22 | 9 | 10 |
| Sample 31 | 14 | 11 | 14 | 10 | 19 | 25 | 23 | 11 | 10 |
| Sample 32 | 14 | 11 | 14 | 12 | 21 | 27 | 22 | 9 | 11 |
| Sample 33 | 14 | 11 | 14 | 12 | 18 | 27 | 22 | 10 | 11 |
| Sample 34 | 14 | 11 | 14 | 12 | 19 | 28 | 22 | 9 | 11 |
| Sample 35 | 15 | 12 | 17 | 13 | 17 | 28 | 23 | 9 | 10 |
| Sample 36 | 14 | 11 | 14 | 12 | 19 | 28 | 22 | 9 | 12 |
| Sample 37 | 14 | 11 | 15 | 12 | 18 | 25 | 23 | 8 | 10 |
| Sample 38 | 14 | 11 | 15 | 12 | 18 | 25 | 23 | 8 | 11 |
| Sample 39 | 14 | 11 | 14 | 12 | 19 | 28 | 22 | 9 | 11 |
| Sample 40 | 15 | 11 | 14 | 12 | 19 | 27 | 25 | 9 | 9 |
| Sample 41 | 14 | 11 | 14 | 12 | 18 | 25 | 23 | 8 | 10 |
| Sample 42 | 14 | 11 | 14 | 11 | 19 | 25 | 23 | 11 | 11 |
| Sample 43 | 14 | 11 | 14 | 12 | 18 | 27 | 22 | 10 | 10 |
| Sample 44 | 14 | 11 | 14 | 10 | 18 | 24 | 23 | 10 | 10 |
| Sample 45 | 15 | 11 | 14 | 12 | 19 | 27 | 25 | 9 | 9 |
| Sample 46 | 14 | 11 | 15 | 12 | 18 | 25 | 23 | 8 | 10 |
| Sample 47 | 14 | 11 | 14 | 12 | 19 | 28 | 22 | 9 | 11 |
|  |  |  |  |  |  |  |  |  |  |
| ***Pnar*** |  |  |  |  |  |  |  |  |  |
| Sample 1 | 15 | 10 | 14 | 10 | 18 | 22 | 24 | 9 | 11 |
| Sample 2 | 15 | 11 | 14 | 12 | 18 | 25 | 22 | 9 | 10 |
| Sample 3 | 14 | 11 | 14 | 12 | 19 | 24 | 22 | 9 | 8 |
| Sample 4 | 14 | 11 | 15 | 12 | 17 | 25 | 23 | 8 | 10 |
| Sample 5 | 14 | 12 | 14 | 13 | 17 | 27 | 23 | 9 | 10 |
| Sample 6 | 12 | 10 | 16 | 9 | 19 | 26 | 28 | 9 | 9 |
| Sample 7 | 14 | 11 | 14 | 11 | 19 | 26 | 25 | 9 | 8 |
| Sample 8 | 14 | 10 | 15 | 11 | 18 | 25 | 23 | 8 | 10 |
| Sample 9 | 12 | 10 | 16 | 9 | 19 | 26 | 28 | 12 | 10 |
| Sample 10 | 14 | 11 | 15 | 13 | 18 | 27 | 21 | 9 | 11 |
| Sample 11 | 12 | 10 | 15 | 13 | 18 | 24 | 24 | 8 | 13 |
| Sample 12 | 14 | 12 | 14 | 13 | 19 | 25 | 24 | 10 | 13 |
| Sample 13 | 14 | 11 | 15 | 12 | 18 | 26 | 23 | 8 | 10 |
| Sample 14 | 14 | 11 | 16 | 11 | 20 | 27 | 22 | 9 | 11 |
| Sample 15 | 12 | 10 | 15 | 13 | 19 | 24 | 24 | 8 | 12 |
| Sample 16 | 15 | 10 | 14 | 11 | 18 | 27 | 25 | 10 | 9 |
| Sample 17 | 14 | 11 | 14 | 11 | 19 | 28 | 22 | 8 | 12 |
| Sample 18 | 14 | 10 | 15 | 12 | 18 | 25 | 23 | 8 | 11 |
| Sample 19 | 15 | 11 | 14 | 12 | 18 | 27 | 25 | 10 | 9 |
| Sample 20 | 14 | 10 | 15 | 12 | 19 | 27 | 25 | 8 | 9 |
| Sample 21 | 15 | 10 | 14 | 11 | 20 | 27 | 25 | 10 | 10 |
| Sample 22 | 14 | 11 | 14 | 12 | 17 | 25 | 24 | 9 | 12 |
| Sample 23 | 15 | 10 | 14 | 12 | 19 | 27 | 25 | 10 | 10 |
| Sample 24 | 15 | 10 | 15 | 12 | 18 | 25 | 23 | 8 | 13 |
| Sample 25 | 14 | 11 | 15 | 12 | 19 | 24 | 24 | 9 | 11 |
| Sample 26 | 13 | 12 | 14 | 13 | 18 | 27 | 23 | 9 | 10 |
| Sample 27 | 14 | 10 | 15 | 12 | 17 | 25 | 23 | 8 | 10 |
| Sample 28 | 12 | 11 | 15 | 13 | 20 | 24 | 24 | 8 | 12 |
| Sample 29 | 14 | 11 | 14 | 11 | 19 | 24 | 23 | 10 | 12 |
| Sample 30 | 14 | 10 | 15 | 12 | 18 | 25 | 23 | 8 | 10 |
| Sample 31 | 15 | 12 | 15 | 13 | 18 | 28 | 24 | 10 | 9 |
| Sample 32 | 14 | 10 | 14 | 12 | 18 | 25 | 23 | 8 | 10 |
| Sample 33 | 14 | 10 | 14 | 12 | 19 | 28 | 22 | 10 | 10 |
| Sample 34 | 14 | 10 | 14 | 9 | 18 | 25 | 23 | 9 | 10 |
